# The effect of views on the COVID-19 pandemic on the development of depressive symptoms in a sample of the elderly

**DOI:** 10.1192/j.eurpsy.2021.1776

**Published:** 2021-08-13

**Authors:** M. Mentis, I. Lamprinakou, C. Marneras, A. Garantoudi, I. Dritsas

**Affiliations:** 1 Education And Social Work Sciences, University of Patras, Greece, Patras, Greece; 2 Health Center, Phaistos Health Center, Moires, Greece; 3 Pathological Clinic, University Hospital of Patras, Patras, Greece; 4 Psychology, Deree College, Athens, Greece; 5 Education And Social Work Sciences, University of Patras, Greece, patras, Greece

**Keywords:** GDS-15, Depression, Elderly, COVID-19

## Abstract

**Introduction:**

The covid-19 virus pandemic is another risk factor not only for the lives of older people, but also for their mental health, as the threat is immediate and intense.

**Objectives:**

The aim of the study was to investigate depression during the pandemic in a population of elderly people over 65 years of age living in the Greek countryside.

**Methods:**

The research was synchronous and was conducted in the autumn of 2020 in Crete. The sample of the study was random and consisted of 200 elderly users of services of the Health Center of the Municipality of Phaistos, Crete. The Geriatric Depression Scale (GDS-15) was used to conduct the study in combination with 24 questions related to pandemic perceptions.

**Results:**

40.5% of the sample were men and 59.5% were women. The mean age was 75.70 years (SD ± 6.29). The mean value of GDS-15 was found to be 5.97 (SD, 3.07), while the comparison of depressive symptoms showed that women, lonely people and the elderly with chronic health problems are more vulnerable to the development of depressive symptoms. In relation to covid-19 and depressive symptoms, there was a positive correlation with twelve factors (p <, 05) with the most important being the fear for their family health, sleep disorder, loneliness and inability to deal with the virus.

**Conclusions:**

The research showed moderate depressive symptoms, while a clear effect of the pandemic due to Covid-19 was found on the emotional mood of the elderly, a fact that makes their psychosocial support necessary.

**Disclosure:**

No significant relationships.

